# An Entity-Based Visual Analytics System Enhancing Medical Expertise Acquisition: Development and Verification Study

**DOI:** 10.2196/83785

**Published:** 2026-05-15

**Authors:** Xiao Pang, Chang Liu, Yan Huang, MingYou Liu, Jiyuan Liu

**Affiliations:** 1 Department of Information Management, State Key Laboratory of Oral Diseases & National Center for Stomatology & National Clinical Research Center for Oral Diseases, West China Hospital of Stomatology, Sichuan University, No. 14, 3rd Section of Ren Min Nan Rd., Chengdu, 610041, China, 86 13880737189; 2 Department of Information and Network Security, Peking Union Medical College Hospital, Chinese Academy of Medical Sciences, Beijing, China

**Keywords:** medical text, medical expertise, illness script, visualization, visual analytics

## Abstract

**Background:**

Acquiring medical expertise from the vast body of medical text is a critical component of medical education. However, the majority of medical knowledge resides in unstructured texts. Data heterogeneity across institutions and strict privacy regulations hinder the use of general-purpose analysis tools. This creates a substantial barrier to the efficient acquisition of expertise for learners.

**Objective:**

This study aimed to design, develop, and evaluate MExplore, an interactive visual analytics system to facilitate the acquisition of medical expertise from unstructured clinical texts.

**Methods:**

We propose a localized, cost-effective workflow for the automatic extraction of medical entities. Building on this workflow, MExplore provides a multilevel visual framework featuring coordinated views for progressive, entity-centered exploration. The system was evaluated through case studies conducted with domain experts, a user study, and semistructured interviews.

**Results:**

The evaluation demonstrated that MExplore significantly enhances the medical expertise acquisition process. Our findings confirm that MExplore provides an effective and interactive approach for structuring complex knowledge, facilitating the construction of illness scripts, and strengthening knowledge retention.

**Conclusions:**

MExplore provides an intuitive and powerful approach for acquiring medical expertise. The results suggest that it effectively supports medical learners in conducting in-depth data exploration and developing robust clinical reasoning skills.

## Introduction

The rapid expansion of medical education has intensified the demand for efficient expertise acquisition [1,2]. Medical expertise is characterized by a highly organized and differentiated knowledge base, technical skills, and perceptual capabilities acquired through extensive domain-related practice [3]. The acquisition of medical expertise is a complex cognitive process that involves constructing abstract knowledge networks and iteratively refining them into narrative structures known as “illness scripts” [4]—cognitive frameworks that link clinical features to diagnoses and enable clinicians to perform complex reasoning tasks efficiently [5]. Traditional learning relies heavily on textbooks and often overlooks real-world medical documents (MDs) such as electronic medical records. Although these documents provide rich, detailed cases for refining illness scripts, their unstructured nature and volume make it difficult for novices to extract and synthesize information.

While large language models (LLMs) have emerged as tools for information synthesis, their utility is limited by practical barriers, such as privacy regulations and computational costs [6]. Furthermore, their direct application in medical education is fraught with risks. Research indicates that LLMs are prone to “deceptive expertise,” generating plausible yet factually incorrect information while failing to recognize their own limitations [7,8]. Overreliance may lead to cognitive offloading, impairing learners’ ability to critically evaluate artificial intelligence–generated outputs [9-12]—a particularly concerning issue in medical education, where independent clinical reasoning is essential. Consequently, there is a pressing need for frameworks that support exploratory learning and active engagement with data, rather than systems that encourage the passive acceptance of output [9,13].

Visual analytics, which integrates human expertise with machine intelligence in a “human-in-the-loop” paradigm, offers a viable approach for such exploratory frameworks. By visualizing complex data, these systems can facilitate the construction of mental models. However, the application of visual analytics to medical expertise acquisition remains underdeveloped. Much of the existing work in this domain has focused on analyzing individual patient electronic medical records to support clinical decision-making for specific cases [14,15], which does not facilitate the higher-level organization of generalizable medical knowledge required for education. Other studies have visualized health care data but often neglect the underlying semantics of the content [16,17], limiting the user’s ability to understand deep clinical relationships. Therefore, a gap exists for a system that can aggregate unstructured text and present it in a semantically meaningful, multilevel structure suitable for learning.

Named entity recognition (NER) serves as a key technique for transforming unstructured text into structured representations. It involves identifying and classifying domain-specific concepts, such as symptoms, diseases, and treatments, within a text [18,19]. While recent advancements in deep learning and transformer-based architectures such as BERT (Bidirectional Encoder Representations From Transformers) have significantly improved the accuracy of extracting these medical entities (MEs) [20-23], the potential of using extracted entities as “anchors” to construct visual knowledge networks remains largely unexplored. Integrating NER with visual analytics offers a novel pathway to transform raw clinical text into structured learning pathways, thereby accelerating the construction of illness scripts.

We present MExplore, a system that leverages NER to extract MEs from unstructured text and organizes them into a coherent visual structure based on a multilevel metaphor. This design allows learners to explore medical knowledge at varying granularities—including MDs, medical paragraphs (MPs), medical entity sets (MESs), and individual MEs. This approach actively engages users in reasoning, reducing the passive learning common in chat-based interfaces [24,25], and reinforcing the cognitive links necessary for robust illness scripts. We validate the system through a multifaceted evaluation involving domain expert case studies, a controlled user study with medical students, and expert interviews to assess clinical validity and user acceptance [26].

## Methods

### Study Design

#### Design Methodology and Workflow

**Figure 1.**
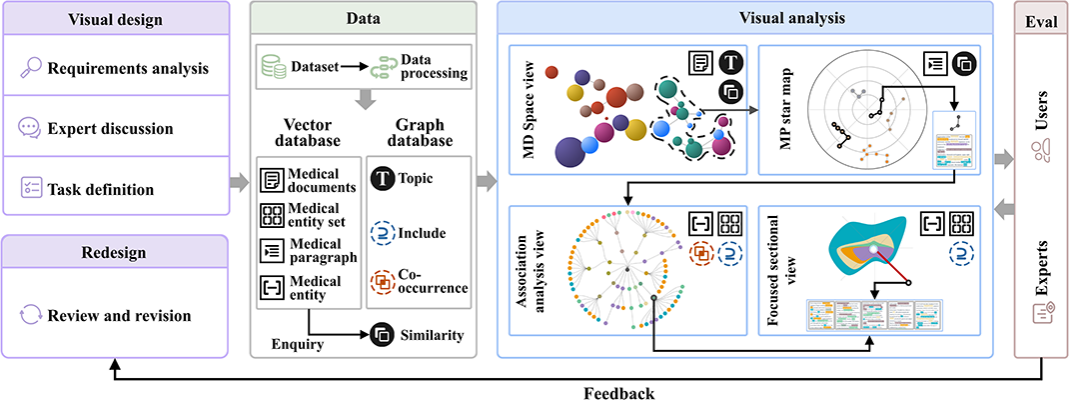
The MExplore workflow encompasses visual design, data processing, storage, and query handling, culminating in multilevel visual analysis. The process incorporates iterative redesigns driven by user and expert feedback. MD: medical document; MP: medical paragraph.

We used a user-centered design process [27], as illustrated in Figure 1. Initially, we collaborated with domain experts to analyze requirements and define specific visual analytics tasks. Guided by these tasks, we established the hierarchy, structural organization, and computational metrics for the textual data. We subsequently designed and developed a multilevel visual analytics framework tailored to these data specifications. Throughout the development lifecycle, we engaged in close collaboration with experts and users, conducting multiple rounds of testing and validation. The feedback and recommendations from these sessions drove iterative redesigns, ensuring continuous system optimization.

#### Requirements Analysis

##### Requirement Elicitation

We collaborated closely with 10 domain experts (E1-E10). E1 and E2 are PhD candidates specializing in medical studies, with 4 and 5 years of focused research experience; E3 is a professor at a medical college with 8 years of teaching experience; E4 is a medical researcher with a decade of research expertise; E5 is a dentist with 10 years of professional practice; E6-E8 are experienced physicians with 5‐10 years of clinical practice; and E9 and E10 are visualization researchers, with 5‐10 years of experience in visual analytics.

Through iterative discussions, the experts (specifically E3-E8) emphasized that acquiring expertise involves refining knowledge into “illness scripts” [4]. These scripts enable clinicians to integrate disease-relevant information and enhance both recall and application [28,29]. To support this, we identified 4 key requirements.

##### R1: Extraction of Core Knowledge Units From Complex Texts

Unstructured medical texts create a high cognitive load for novice learners due to their density and complexity [30]. Novices often face a “reading bottleneck” [31] where resources are consumed by decoding information rather than deep comprehension. Therefore, it is essential to extract core knowledge units and filter out redundant information to mitigate extraneous cognitive load [32,33].

##### R2: Organization of Text With Varying Knowledge Density to Support Gradual Exploration

Presenting excessive details simultaneously causes cognitive overload [32,34]. Experts suggest that information should be presented at appropriate levels of abstraction [35]. Medical texts must be restructured with varying knowledge densities to support an incremental learning process, moving from high-level overviews to granular details [36].

##### R3: Revealing the Interconnections Between Knowledge Units

Rote memorization is insufficient for long-term retention [37]. Learners must actively integrate new information with existing schemas [38]. Consequently, there is a need to visually reveal the logical interconnections and inclusion relationships between knowledge units to support structural understanding.

##### R4: Focused Analysis of the Key Knowledge Units

Effective knowledge acquisition relies on identifying and analyzing key knowledge units that serve as cognitive “anchors” [39,40]. Focused analysis of these units not only deepens understanding but also facilitates the establishment of broader connections within the medical field [40], thereby reinforcing long-term retention.

### Visual Analytics Tasks

Based on the requirements (R1-R4), we derived 4 visual analytics tasks (T1-T4) to guide the system implementation.

#### T1: Extract MEs From Real-World Medical Texts

The system must automatically extract MEs from raw medical texts and categorize them. This transforms unstructured data into discrete, manageable knowledge units (R1), which serve as the foundational elements for visualization.

#### T2: Support for Cascading Visual Analysis and Exploration of Texts With Varying Knowledge Densities

The system should organize extracted data into a hierarchical structure. It must provide cascading views that allow users to transition seamlessly from a high-level thematic overview to coarse-grained paragraph relations, and finally to fine-grained entity details. This capability facilitates incremental exploration and understanding of complex medical knowledge (R2).

#### T3: Establishing a Clear Structure for Association Analysis

The system needs to construct and visualize a clear mapping of relationships, specifically the inclusion of MEs within paragraphs and the co-occurrence of elements. This visualization should clarify how knowledge units are structurally connected (R3).

#### T4: Support for Entity-Centric Pattern Analysis

The system must provide interaction mechanisms to select a specific ME or MES as a focal point. Upon selection, the visualization should be dynamically reconfigured to display the distribution, composition, and associated context centered around the target knowledge unit (R4).

### Dataset Construction

#### Data Source

In this paper, we analyze and process 2 realistic datasets. The first dataset is the Chinese Biomedical Language Understanding Evaluation (CBLUE) provided by the Key Laboratory of Computational Linguistics (Peking University, China) [41]. CBLUE is a comprehensive benchmark compiled from authoritative medical textbooks and clinical practice records. The dataset contains approximately 96,000 MDs, covering a wide range of more than 500 common diseases. To ensure high quality, the MEs within the dataset were meticulously annotated by medical experts. These MEs are categorized into 9 classes on the basis of Chinese ME annotation standards [41]: disease (dis), clinical symptoms (sym), drugs (dru), medical equipment (equ), medical procedures (pro), body (bod), medical examination items (ite), microorganisms (mic), and department (dep).

The second dataset, the Medical Text of West China School (Hospital) of Stomatology (MWCSS), was collected between 2023 and 2025 and provided by the West China School (Hospital) of Stomatology (Sichuan University, China). This dataset contains a large volume of unstructured text data from clinical practice. It includes approximately 100,000 MDs, covering various facets of medical processes, such as clinical diagnoses, treatment processes, specialist examinations, auxiliary examinations, treatment plans, interventions, and drug instructions.

#### ME Extraction Comparative Analysis

The accuracy of ME extraction is critical to the effectiveness of data processing and visual analytics. Therefore, we systematically assessed the performance and computational resource demands of several models for ME extraction.

Due to strict data confidentiality protocols, which preclude the transmission of data to external servers, models must be deployable locally. In light of the practical limitations of resource-constrained environments, and based on the latest benchmark list from FlagEval [42], we selected high-performing, open-source LLMs for evaluation: DeepSeek-70B and Qwen-32B. Additionally, we included MacBERT, an enhanced BERT model with a novel masked language modeling correction pretraining task [43], which has been identified as the top-performing BERT-based model according to the CBLUE benchmark, for comparison.

Fine-tuning and inference were performed via two NVIDIA A10 GPUs or a single NVIDIA RTX 4060 Ti GPU for MacBERT. The fine-tuning parameters were as follows: learning rate of 3e-5, 2 epochs, batch size of 16, and warm-up ratio of 0.1. For DeepSeek-70B and Qwen-32B, both models required two A10 GPUs and were instruction-tuned via 4-bit quantized models with QLoRA (Quantized Low-Rank Adaptation) [44] (LoRA [Low-Rank Adaptation] settings: *r*=16, *α*=64, dropout=0.05). All the fine-tuning parameters and LLM prompt words were selected based on benchmark tests [41] and relevant comparative experiments [45] and have been validated. The computational resource consumption for each model is summarized in Table 1.

**Table 1.** Computational resource consumption comparison.

Model	GPU configuration (NVIDIA)	Fine-tuning time (hours)	Inference time (hours)
DeepSeek-70B	2 A10	167.23	134.27
Qwen-32B	2 A10	101.46	83.58
MacBERT	2 A10	0.77	0.18
MacBERT	1 RTX 4060 Ti	5.16	0.35

The results highlight a significant disparity in resource demand. While LLMs (DeepSeek-70B and Qwen-32B) require substantial fine-tuning and inference time on dual A10 GPUs, MacBERT demonstrates remarkable efficiency, completing the same tasks in a fraction of the time. Notably, MacBERT’s inference speed on consumer-grade hardware (a single RTX 4060 Ti) suggests that it is highly suitable for local deployment in practical medical environments where high-end computational clusters may not be available.

Table 2 summarizes the performance metrics for each model. Fine-tuning consistently improved performance across all the models. Notably, the fine-tuned MacBERT model achieves the highest *F*_1_-score (0.605), surpassing both LLMs, and its precision approaches human annotation levels [41].

**Table 2.** Performance comparison of models for medical entity extraction.

Model	Type	Precision	Recall	*F*_1_-score
DeepSeek-70B	Base	0.548	0.487	0.516
DeepSeek-70B	Fine-tuned	0.593	0.521	0.535
Qwen-32B	Base	0.554	0.491	0.525
Qwen-32B	Fine-tuned	0.608	0.542	0.561
MacBERT	Base	0.595	0.549	0.571
MacBERT	Fine-tuned	0.636	0.579	0.605

This analysis demonstrates that MacBERT not only delivers the best performance after fine-tuning but also requires significantly fewer computational resources than the LLMs. MacBERT, therefore, emerges as the optimal choice for ME extraction tasks. This conclusion aligns with the literature [46], which emphasizes that while LLMs excel in generative tasks, BERT-based models retain distinct advantages in NER and other specialized domains [47] and sentiment analysis [48].

Beyond common medical conditions, the system’s robustness concerning rare diseases and less common terminology warrants further discussion. Although our evaluation used a dataset of common diseases—where MacBERT demonstrated strong performance—recent research [49] suggests that the performance landscape may shift when encountering “long-tail” medical data. Specifically, while fine-tuned BERT-based models generally maintain overall superiority, LLMs can exhibit superior capabilities in identifying rare disease entities in few-shot settings. Therefore, for specialized tasks focusing on rare diseases where relevant samples and sufficient computational resources are available, LLMs may be used to achieve higher precision. Nevertheless, within our current visual analytics framework centered on general-purpose unstructured medical text, MacBERT provides the most reliable performance-to-cost ratio.

For NER requiring deep semantic insight, language-specific pretrained models often outperform general multilingual models [50]. In this study, we use MacBERT—the top-performing model on the CBLUE dataset—to maximize extraction accuracy for Chinese corpora. Notably, the underlying pipeline remains highly adaptable; the base encoder can be seamlessly replaced (eg, substituting BioBERT [51] for MacBERT) to process English or other language datasets.

#### Data Processing

**Figure 2.**
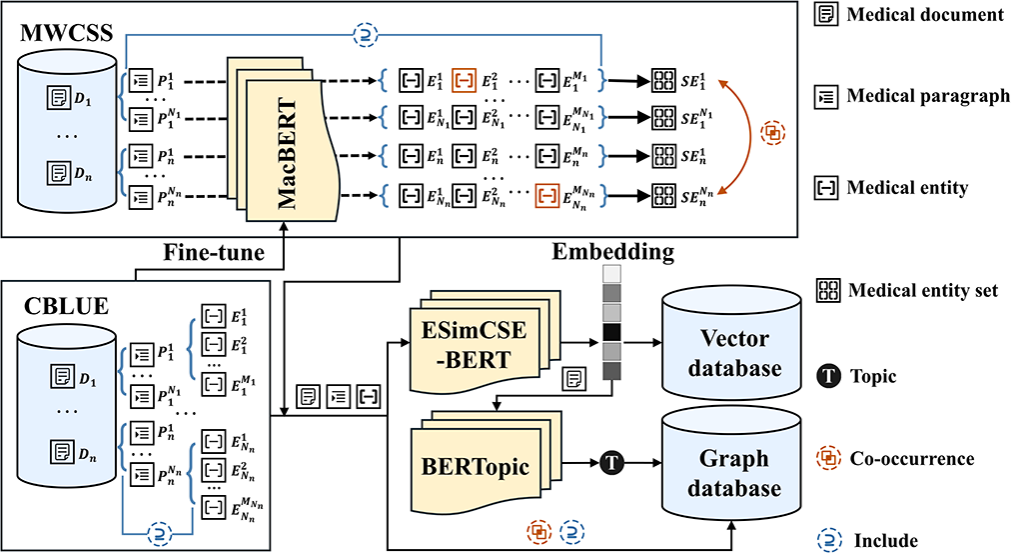
Pipeline of data processing. First, MacBERT was fine-tuned using labeled data from CBLUE. Individual MPs within the MDs are processed via the fine-tuned MacBERT model to extract MEs, which are subsequently organized into MESs. These texts are input into ESimCSE-BERT to generate embeddings, which are then stored in a vector database. The MD embeddings serve as the basis for topic clustering in BERTopic. The resulting topics of MDs, along with all medical texts and their inclusion and co-occurrence relationships, are then structured into a graph and stored in a graph database. CBLUE: Chinese Biomedical Language Understanding Evaluation; *D*: medical document; *E*: medical entity; MD: medical document; ME: medical entity; MES: medical entity set; MP: medical paragraph; MWCSS: Medical Text of West China School (Hospital) of Stomatology; *P*: medical paragraph; *SE*: medical entity set.

Figure 2 shows the pipeline of data processing. The process can be described as follows.

##### Step 1: ME Extraction and Structuring

According to the results of the ME Extraction comparative analysis, we used the MacBERT model fine-tuned with the CBLUE dataset to extract MEs from MWCSS, using MP of MD *D_i_* as the extraction unit, thereby constructing MESs.

##### Step 2: Vectorization

All the text from CBLUE and MWCSS, including MDs, MPs, MESs, and MEs, is processed via ESimCSE-BERT [52]—an efficient and improved unsupervised sentence embedding method that generates high-quality sentence vectors.

##### Step 3: Topic Clustering

The high-quality MD vectors from step 2 were input into the BERTopic model. By leveraging these superior embeddings, the clustering process produces topic assignments for each MD. The results yielded a topic diversity score [53] of 0.975, indicating a high degree of distinctness among the generated categories. To further validate the clinical utility, a 5-point Likert scale assessment was conducted by medical experts (E3, E4, E6, E7, and E8) to evaluate the effectiveness of the generated topics and keywords (Table 3). Following the evaluation framework proposed in [54], the results suggest that the interpretability, distinctiveness, and relevance of the topic clusters are at a high level. These findings further corroborate the superiority of transformer-based topic models as discussed in [54].

**Table 3.** Evaluation of topic clustering (1‐5 Likert scale).

Evaluation	Explanation	Score, mean (SD)
Interpretability	How easily a human can assign a coherent meaning to the topic.	4.3 (0.57)
Distinctiveness	How well the topic is differentiated from other topics.	4.4 (0.41)
Relevance	How well the topic captures meaningful domain content.	4.6 (0.22)

##### Step 4: Relationship Mapping and Storage

To support complex analytical queries, we implemented a dual-storage strategy addressing the inherent relational complexity and interconnectedness of medical data.

###### Semantic Storage in the Vector Database

All textual elements (MDs, MPs, MESs, and MEs) are embedded into high-dimensional vectors and stored in a vector database. This infrastructure leverages various optimization algorithms to enable the efficient execution of similarity-based queries and fast calculations across datasets in the tens of millions [55].

###### Structural Storage in the Graph Database

Each text unit is modeled as a vertex, with MD vertices carrying the topic attributes derived from clustering. The inclusion relationships between texts and the co-occurrence of MEs within MESs are represented as edges. This graph structure is stored in a graph database to facilitate high-performance queries across complex entity relationships [56]. The scalability and query efficiency of this graph-based approach have become increasingly evident with larger datasets [57].

### Visual Analytics Systems Development

#### System Overview and Workflow

**Figure 3.**
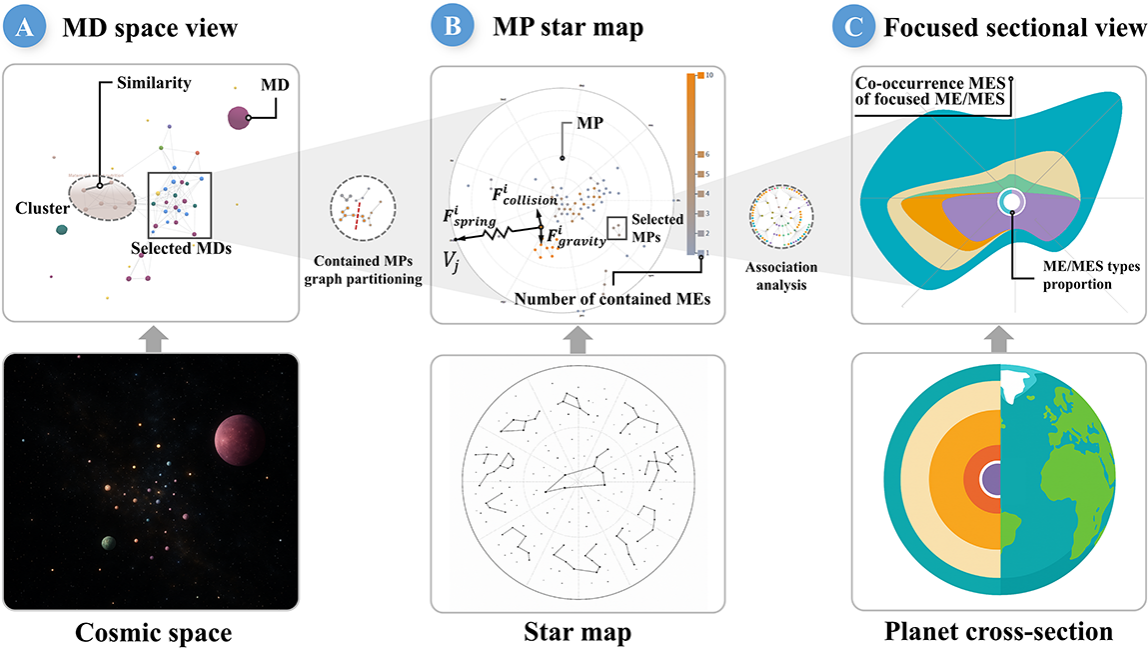
The MExplore framework: (A) the MD space view, inspired by cosmic space, where users select MDs and construct and partition graphs of the contained MPs; (B) the MP Star map, inspired by the star map, where users choose corresponding MP subgraphs and perform relational analysis; (C) the focused sectional view, inspired by the planet cross-section, which enables detailed, focused analysis of the selected data. *F*_*collision*_: the collision force; *F*_*gravity*_: the gravity force; *F*_*spring*_: the spring force; MD: medical document; ME: medical entity; MES: medical entity set; MP: medical paragraph.

Building upon the constructed dataset, we developed MExplore, a multilevel visual analytics system designed to facilitate progressive exploration and cascading visual analysis (T2). The system adopts a 3-tiered metaphor—cosmic space, star map, and planet cross-section—which inspires the design of the MD space view (Figure 3A), the MP star map (Figure 3B), and the focused sectional view (Figure 3C). We use a hierarchical layout that systematically guides users from macrolevel document retrieval to microlevel detail exploration.

The analytical workflow follows a top-down trajectory (Figure 3), starting from the MD space view, where users filter and define a subset of MDs as the basis for all subsequent analysis based on cluster and spatial distributions. Once the analytical scope is established, the system transitions to the MP star map, enabling users to identify structural patterns and semantic clusters within MP subgraphs. From there, users can incorporate specific subgraphs into the association analysis view to reveal intricate relationships between MESs and MEs. For the most granular inquiry, users can select individual nodes within this view to generate a corresponding focused sectional view, facilitating a deep dive into the target knowledge unit’s context.

By structuring the analysis as an interactive, stepwise process, MExplore allows users to progressively increase analytical granularity without losing global contextual coherence [58]. Furthermore, the design leverages grounded metaphors to map complex information onto pre-existing cognitive schemas [59], effectively mitigating the visual complexity encountered by the user and enhancing memory encoding for knowledge retention [60]. Ultimately, this multitiered approach facilitates a continuous analytical loop.

#### MD Space View

**Figure 4.**
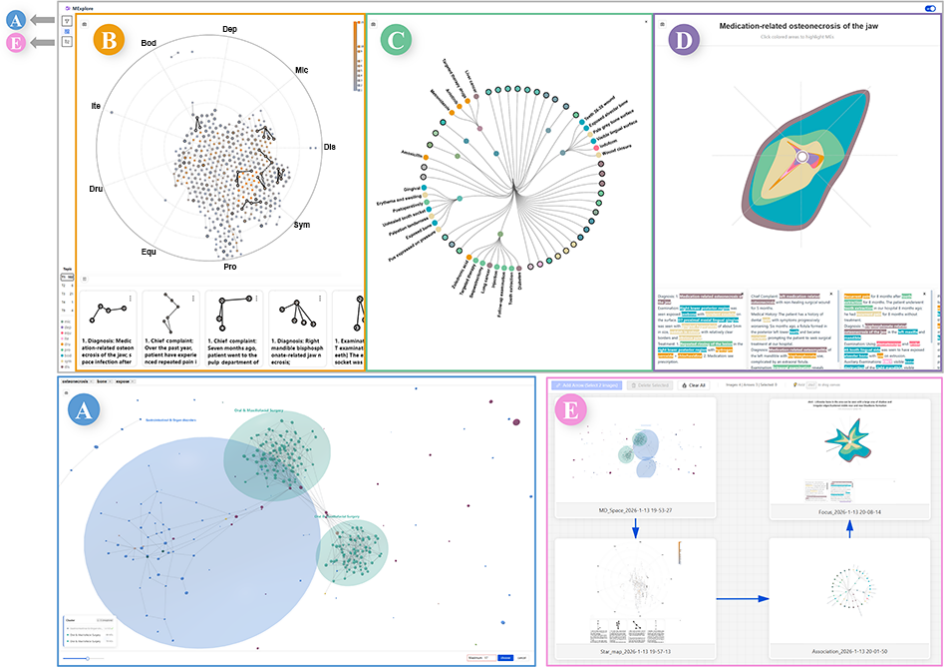
The MExplore system: (A) MD space view; (B) MP star map; (C) association analysis view; (D) focused sectional view; (E) provenance view. MD: medical document; MP: medical paragraph.

As shown in Figure 4A, upon entering keywords, the system executes a full-text vector search to retrieve relevant MDs. The retrieved MDs are mapped into a 3D force-directed graph where each MD is represented as a planet—its size encodes text length, and its position is determined by semantic similarity. Interdocument links visualize this similarity. Users can adjust a threshold slider to filter connections by similarity, aggregating MDs into topic-based clusters represented as nebulae labeled with topic titles. This layout emphasizes core structural relationships and allows users to follow a progressive approach starting from these nebulae, clicking a specific nebula to enter Focus Mode to explore individual MDs in detail. This interaction mechanism leverages spatial proximity to facilitate the efficient identification of areas of interest [61].

The colors of the nebulae and their constituent planets represent their respective topics. This color encoding is discarded upon transitioning to detailed analysis views, preventing semantic interference between the MD topic and ME attributes.

To facilitate comprehensive discovery, a guide for unexplored nebulae is displayed in the lower-left corner, allowing for thorough exploration and rapid focusing by clicking. Simultaneously, the panel below ranks MDs by vector similarity scores to support instant focused viewing and prevent users from overlooking important MDs. Throughout this process, users iteratively select MDs to build an analytical collection, which serves as the foundation for all subsequent analysis views.

#### MP Star Map

Relying solely on full-text embedding similarity can introduce bias, as document representations may disproportionately reflect longer sections while overshadowing concise but critical information. For instance, detailed medical histories in outpatient records can overshadow concise diagnosis sections during similarity computation.

**Figure 5.**
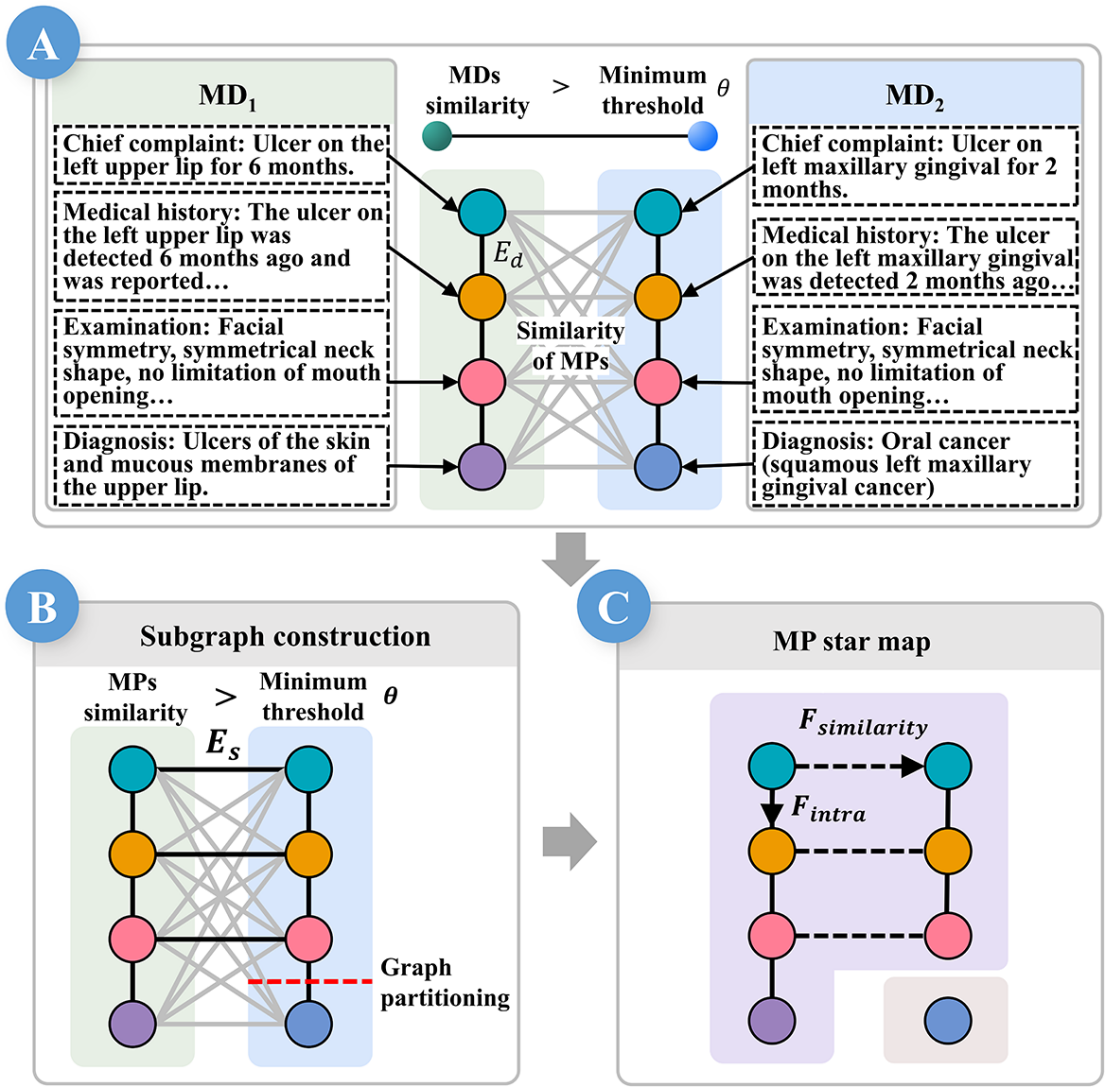
For detailed analysis, MDs are decomposed into MPs. (A) MPs are represented as vertices, with *E_d_* connecting MPs within the same MD, and the similarity of MPs across connected MDs is computed. (B) MPs whose similarity is greater *θ* than are connected by *E_s_*, and the graph is partitioned by KaFFPa. (C) The partitioned subgraphs are visualized via the constellation metaphor, where *E_d_* are shown as lines and the MPs are shown as stars. Each star is subject to *F*_*intra*_ from *E_d_* and *F*_*similarity*_ from *E_s_*. MD: medical document; MP: medical paragraph.

To mitigate this, MDs are decomposed into MPs, each modeled as a graph vertex. Intradocument edges *E_d_* connect the MPs within the same MD. Cross-document MP pairs (Figure 5A) exceeding the similarity threshold set in Figure 4A are linked by similarity edges *E_s_*. The KaFFPa algorithm [62] then partitions the graph, segregating semantically distinct MPs into subgraphs (Figure 5B).

Each resulting subgraph is visualized as a constellation in the MP star map (Figure 4B), the MPs are depicted as stars, and *E_d_* is rendered as a constellation line, whereas *E*_*s*_ is omitted to reduce clutter but is retained as latent factors influencing the layout, the similarity-based gravitational force *F*_*similarity*_, and the structural gravitational force *F*_*intra*_ (Figure 4C). The combined gravitational force of the star *i* is calculated as follows:


(1) $$F_{gravity}^{i}=F_{intra}^{i}+F_{similarity}^{i}$$

(2) $$F_{similarity}^{i}=\sum_{\left(i,j\right)}^{similarity_{i}^{j}§gt;\theta }(\left\|F\right\|\cdot similarity_{i}^{j})\;\cdot \hat{d_{i}^{j}}$$

where *F*_*intra*_ is the link force connecting the MPs within the same MD, with a magnitude of unit force ‖F‖, *θ* is the threshold set by the user, and $similarity_{i}^{j}$ is the similarity between MP *i* and MP *j*. ${\hat{d}}_{i}^{j}$ is the unit vector of the force direction from MP *i* to MP *j*.

In addition, each star *i* is subjected to spring forces $F_{spring}^{i}$ from 9 ME-type poles uniformly distributed on the circular boundary of the star map:


(3) $$F_{spring}^{i}=\sum_{j=1}^{9}\frac{num_{i}^{j}}{num_{i}}\cdot \left\|F\right\|\cdot {\hat{d}}_{i}^{j}$$

where $num_{i}^{j}$ is the number of MEs of type *j* in MP *i*, and *num_i_* is the total number of MEs in MP *i*. ${\hat{d}}_{i}^{j}$ is the unit vector of the force direction from MP *i* to *V_j_*.

To prevent occlusion, each star *i* is subject to the collision force $F_{collision}^{i}$ of stars that may overlap it. The final combined force $F_{collision}^{i}$ is calculated as follows:


(4) $$F_{combined}^{i}=F_{gravity}^{i}+F_{spring}^{i}+F_{collision}^{i}$$

This multiforce design achieved semantically coherent clustering (*F_spring_*), representation of cross-document similarity and structural relationships (*F_gravity_*), and clear visualization (*F_collision_*) (Figure 4B). Stars within the same constellation are assigned a uniform color, allowing users to quickly identify them even on a densely populated map. This color is modulated by a sequential luminance gradient [63] to encode the star count: brighter constellations denote a greater number of constituent stars, enabling users to efficiently pinpoint pattern-dense regions. The luminance of star borders encodes the ME count, providing information density cues (T2). To preserve macrocontext awareness during the analysis of MPs and subsequent tasks, a topic navigation list at the bottom of the sidebar (Figure 4) visualizes the topic information of selected MDs. In the MP star map and subsequent views, users can click on MP/MES nodes or document cards to highlight the topic of the corresponding document. This mechanism enables on-demand backtracking, ensuring users remain oriented within the macrolevel topic context. This design facilitates progressive schema construction [33,34] and supports iterative exploratory learning.

#### Association Analysis View

Leveraging the concepts within MPs to create visual representations of hierarchical structures can significantly increase the efficiency of knowledge navigation [64,65]. Therefore, we extract MEs from each MP (T1), compose the MES, and visualize the resulting data via a radial dendrogram. Compared with alternative text visualization methods, such as word clouds or Sankey diagrams, this approach effectively captures both inclusion and relational connections among elements while accurately displaying hierarchical structures. To further enhance visualization, we propose an algorithm that groups MESs containing shared MEs into the same tree branch, thereby emphasizing co-occurrence relationships between the MESs (T3). The algorithm is outlined in Textbox 1.

Textbox 1. **Algorithm for tree construction.**

**Input:**

1. Root node of the tree: $r_{tree}$
2. Set of nodes to be added: $N_{add}$

**Output:** Root node of the tree: $r_{tree}$

1. **for** each node $n_{i}$ in $N_{add}$ **do**
2.
$C=GetMEs(n_{i})$

3.
$N,M_{checked}=TraverseTree(r_{tree},C)$

4. end for
5.
$N_{childern}⟵N\cup M_{checked}$

6. **for** each node $n_{i}$ in N **do**
7.
$RemoveFromTree(r_{tree,n_{i}})$

8. **end for**
9. **if** $L_{N}§gt;1$ **then**
10.
$n_{f}⟵CommonFatherNode(r_{tree},N)$

11. **if** $n_{f}$ is not $\left(null\;or\;r_{tree}\right)$ **then**
12.
$n_{f}\cdot Children⟵N_{children}$

13. **else**
14.
$r_{tree}\cdot children⟵r_{tree}\cdot Children\;\cup \;N_{children}$

15. **end if**
16. **end if**
17. **return** $r_{tree}$

For each MP, the extracted MEs form an MES, which acts as a node. The newly added node set *N_add_*, along with the root node of the tree *r_tree_*, is provided as input. For each node *n_i_* in *N_add_*, the algorithm retrieves its associated MEs via *GetMEs*(*n_i_*) function. It then traverses the tree through *TraverseTree* to identify the set *M_checked_* of co-occurring MEs, along with the node set *N* containing these MEs. They are all grouped into the set *N_children_*, which are subsequently added as children to the appropriate parent nodes.

If multiple nodes intersect, their common ancestor is identified by *CommonFatherNode*, and the new nodes are added as children of this ancestor. The nodes are added directly to the root if no common ancestor is found. The process concludes by returning the updated tree structure.

Based on the algorithm described above, the association analysis view is constructed (Figure 4C). To manage the cognitive load and ensure visual consistency, the system uses a synchronized color encoding strategy and a progressive disclosure mechanism. Categorical colors are assigned to ME nodes based on their entity types, while for MES nodes, the color is derived from a weighted mixture of its constituent ME types. To maintain cross-view coherence, this encoding scheme is applied uniformly across both the association analysis and focused sectional views, supported by a persistent legend in the sidebar that ensures color-to-type mappings remain readily accessible throughout the analytical process.

The risk of information overload is further mitigated by initially displaying only the aggregated MES nodes, allowing users to perceive the high-level relational structure without being overwhelmed by individual entities. Users can then interactively expand specific MES nodes to reveal their constituent MEs as independent child nodes. This progressive disclosure strategy enables users to manage information density dynamically according to their analytical needs.

These design choices facilitate the perception of complex hierarchical and relational patterns, thereby supporting schema construction and clinical reasoning by externalizing the learner’s cognitive processes [66,67]. Furthermore, the system architecture supports the dynamic addition of new MPs, enabling real-time reconstruction of the association tree. This capability allows seamless integration of new clinical information into the existing cognitive framework of the user, promoting iterative, exploratory learning and long-term memory retrieval [68].

#### Focused Sectional View

To enable granular analysis of a specific ME or MES, the focused sectional view (Figure 4D) uses a “planetary layer” metaphor to organize complex co-occurrence data. The central ring functions as a donut chart that encodes the proportional distribution of ME classifications. If the analytical focus is a single ME, this layer identifies its specific classification. Surrounding this core, the mantle layer uses a polar-axis-aligned area chart to visualize co-occurring MESs. In this arrangement, each radial axis represents a distinct MES, with the axis height being $N_{i}^{k}*h_{t}$, which represents the number of MEs belonging to class *k* in the *i*th MES. To emphasize semantic continuity across the dataset, areas representing the same ME classification are connected across adjacent axes. Compared with traditional area or stream charts, this radial layout effectively portrays the compositional richness and semantic associations of the target entity within a compact structural signature.

To bridge the gap between visualization and raw text, the system allows users to click a radial axis to query the underlying database and retrieve the corresponding MD, which is then displayed as a document card in the panel below. This design enables users to revert to the original evidence for detailed, word-by-word analysis. To further prevent highlighting overload in the text view, the system implements a selective filtering strategy. While the card highlights various extracted MEs, users can click specific semantic regions in the polar area chart to isolate and highlight only the ME category of interest. This interaction triggers the document card to automatically scroll to the relevant text segment, enabling an efficient and focused review of the original evidence without cognitive distraction.

The resulting structural signatures illustrate the contextual association patterns unique to each focused ME/MES (T4). Furthermore, the mantle’s radial patterns facilitate the interactive comparison of MESs with similar ME configurations. This comparative analysis fosters deeper cognitive engagement and more enduring conceptual impressions, supporting the learner’s ability to internalize complex clinical relationships [67].

#### Provenance View

To facilitate knowledge retention and structure the analytical process, we developed the provenance view (Figure 4E). This view enables users to capture high-fidelity snapshots from any analytical view on demand. These snapshots function as interactive nodes on a scalable, zoomable canvas, allowing users to freely organize layouts and establish logical links between visual findings. By externalizing exploration history into an explicit visual learning path, users can revisit and inspect detailed analytical states at any time, effectively transforming ephemeral interactions into organized knowledge assets.

### Evaluation Design

#### Evaluation Overview

To comprehensively evaluate the usability, effectiveness, and expertise acquisition capabilities of MExplore, we used a mixed methods evaluation strategy comprising three components: (1) case studies with domain experts to assess the practicality, (2) a comparative user study with medical students to quantify learning outcomes, and (3) semistructured interviews with experts to evaluate usability and future potential of the system.

#### Implementation and Apparatus

As a web-based visual analysis system, MExplore was developed using the d3.js and Django frameworks. A Microsoft Windows platform with a 2.71 GHz Intel Core i5-7300 CPU and 8 GB of memory was used as the front-end page server. The evaluation experiments were performed via a Google Chrome web browser.

#### Case Study

The case study can demonstrate feasibility and usability in performing real-world tasks [69]. Therefore, we conducted case studies with domain experts (E3 and E5) to evaluate MExplore within authentic analytical scenarios. Through extensive discussion, the experts identified three critical stages of medical expertise acquisition as core tasks: (1) the discovery of areas of interest, (2) the association analysis, and (3) the construction of illness scripts. The session commenced with a 20-minute tutorial on MExplore’s features, followed by 1 hour of autonomous exploration focused on the predefined tasks. To capture nuanced qualitative feedback, a think-aloud protocol was used, which encouraged participants to externalize their thought process, ask questions, or provide verbal comments throughout the session.

#### User Study

##### Study Overview

The primary objective of the user study was to quantitatively assess the effectiveness of MExplore in facilitating the acquisition of medical expertise. We adopted illness script construction as the evaluative metric to operationalize the measurement of expertise [4]. The experimental design and comparative study protocols were informed by established methodologies in visual analytics and medical education research [15,70-72].

##### Participants

We recruited 20 second-year undergraduate medical students (10 male and 10 female). None of the participants had systematic prior knowledge of the specific diseases selected for the task, thereby minimizing potential confounding effects from background knowledge.

##### Tasks and Materials

Three diseases—oral candidiasis (D1), meningitis (D2), and herpes zoster (D3)—were selected from a list of diseases for which illness scripts could be activated [73]. The participants had not studied these diseases systematically beforehand, thereby eliminating the potential confounding effect of prior knowledge. Following prior studies [70], the task began by providing the participants with a brief description of typical cases for each disease, including relevant medical history and examination results, excluding the disease name. Based on this information, participants were asked to identify the disease and complete an illness script template, the information of which was divided into 3 categories: enabling conditions (EC), fault (FT), and consequences (CQ) [28,73].

##### Experimental Procedure

Participants were randomly assigned to two balanced groups (n=10 each, 5 males and 5 females). The experimental group (MEX) used MExplore to complete the tasks. The control group (OTH) was permitted to use external resources (eg, textbooks, case retrieval systems, and LLMs) but was restricted from using MExplore. Before the main task, the MEX group received a detailed introduction and a 20-minute training session to familiarize themselves with the visual views.

##### Metrics

Performance was evaluated based on the accuracy and time taken to form illness scripts. Accuracy was scored by comparing responses to a standardized answer key established by the expert panel. Each correct information unit was awarded one point. The total score was normalized to a percentage [74]. Furthermore, to assess the long-term impact of MExplore on expertise retention, a follow-up test was conducted 2 weeks after the initial experiment. The participants were asked to fill out the answer sheets again based solely on recall, without access to the system or external aids [75].

##### Statistical Analysis

All analyses were conducted via Python (version 3.10). The performance was evaluated via 3 metrics: accuracy, retention accuracy, and completion time. Both accuracy and retention accuracy are expressed as percentages. Independent sample *t* tests were used to compare the mean differences between the MEX and OTH groups. Two-sided *P*<.05 was considered statistically significant.

### Expert Interview

We conducted semistructured interviews with domain experts (E1-E10); such qualitative feedback is essential to verify whether users benefit from the system support in their specific domain problems [27]. Each expert subsequently completed a 10-item standardized questionnaire (Table 4) via a 5-point Likert scale to assess their attitudes toward MExplore.

**Table 4.** Expert interview questionnaire^a^.

Question	Question content
Q1	MExplore is very easy (difficult) to learn.
Q2	MExplore is very easy (difficult) to use.
Q3	The visual design of MExplore is easy (difficult) to understand.
Q4	The visual interactions of MExplore are easy (difficult) to use.
Q5	I am very willing (unwilling) to use MExplore in exploring and acquiring medical expertise.
Q6	Using MExplore, I can (cannot) efficiently identify core knowledge units within complex medical texts. (R1)
Q7	Using MExplore, I can (cannot) explore medical texts in a gradual, structured manner. (R2)
Q8	Using MExplore, I can (cannot) identify and analyze the interconnections between knowledge units. (R3)
Q9	Using MExplore, I can (cannot) focus on and analyze key knowledge units in detail. (R4)
Q10	MExplore can (cannot) help me construct a comprehensive and reliable illness script.

a Q1-Q5 focus on assessing the system performance of MExplore, Q6-Q9 evaluate whether the key analysis requirements (R1-R4) are satisfied, and Q10 is the overall objective.

### Ethical Considerations

The study received ethics approval from the Institutional Review Board of West China Hospital of Stomatology, Sichuan University (ethical approval number: WCHSIRB-D-2024‐335; approval date: August 9, 2024). Due to its retrospective nature, this study required no informed consent. All collected data were fully anonymized, and no personally identifiable information was included. No financial or material incentives were provided to the participants.

## Results

### Case Study

#### Case 1: Identification and Exploration of Areas of Interest

**Figure 6.**
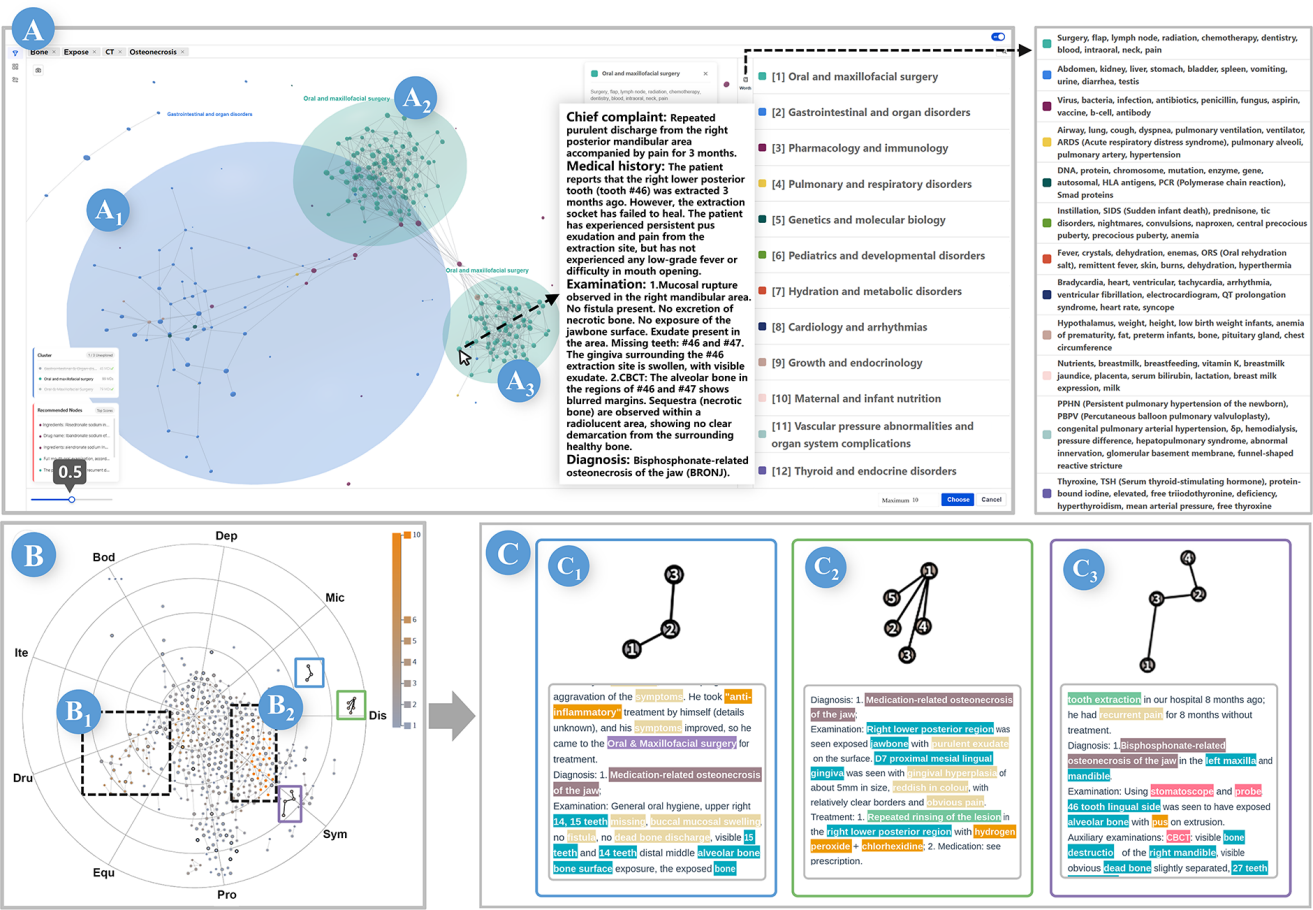
(A) Topics and distribution of keyword-related MDs, with a connection threshold set to 0.5. (B) Distribution of MP subgraphs within the MDs in A2. (C) The original MD texts of the selected MP subgraphs, with border color annotations indicating their corresponding constellation in the star map. MD: medical document; MP: medical paragraph.

The case study begins with a real scenario with exposed bone in the facial area. A CT scan confirmed the presence of osteonecrosis. The experts entered the keywords *bone*, *expose*, *CT*, and *osteonecrosis* in MExplore. This query generated an MD space where, upon setting the similarity threshold to 0.5, a distinct structure comprising 3 nebulae (A1, A2, and A3) emerged (Figure 6A).

The color of nebula A1 corresponded to topic 2 (*Gastrointestinal and Organ Disorders*), whereas A2 and A3 aligned with topic 1 (*Oral and Maxillofacial Surgery*). Consistent with the patient’s clinical presentation in the maxillofacial region, the experts disregarded A1 to concentrate their analysis on A2 and A3. A detailed exploration of A3 revealed that it contained MDs describing symptoms highly congruent with the patient’s condition. Notably, these MDs demonstrated strong connectivity to nodes in topic 3 (*Pharmacology and Immunology*), which also appeared in the recommendation list, confirming their high relevance to the search query. Consequently, E3 selected these MDs, along with those in A3, and transitioned them to the MP star map. The maximum size of the subgraph was set to 10, which was determined during the experts’ free exploration and proved effective for segmenting common MDs.

In the MP star map (Figure 6B), E5 first examined subgraphs close to *dis* and *sym* to identify the specific conditions. Three subgraphs were selected to review the text. After these MDs were compared with the original MDs (Figure 6C), the experts concluded that the target disease was medication-related osteonecrosis of the jaw (MRONJ). E5 commented, “Compared with standard search engines, MExplore facilitates the discovery of trustworthy MDs. The structured visual representations of knowledge units enable users to identify the target disease through interactive reasoning rather than randomly searching through uncertain sources.”

#### Case 2: Association Analysis and Identification of Key Factors

Upon identifying the target disease, the experts conducted an association analysis to determine the key factors related to the illness script. As defined by Feltovich and Barrows [29], illness scripts consist of 3 components: EC, FT, and CQ. In this case, the disease’s name suggested a connection to the medication, leading the experts to select subgraphs that distribute bias toward *dru* (Figure 6B1). Additionally, the subgraphs adjacent to the target disease with a high total number of MEs were selected (Figure 6B2).

**Figure 7.**
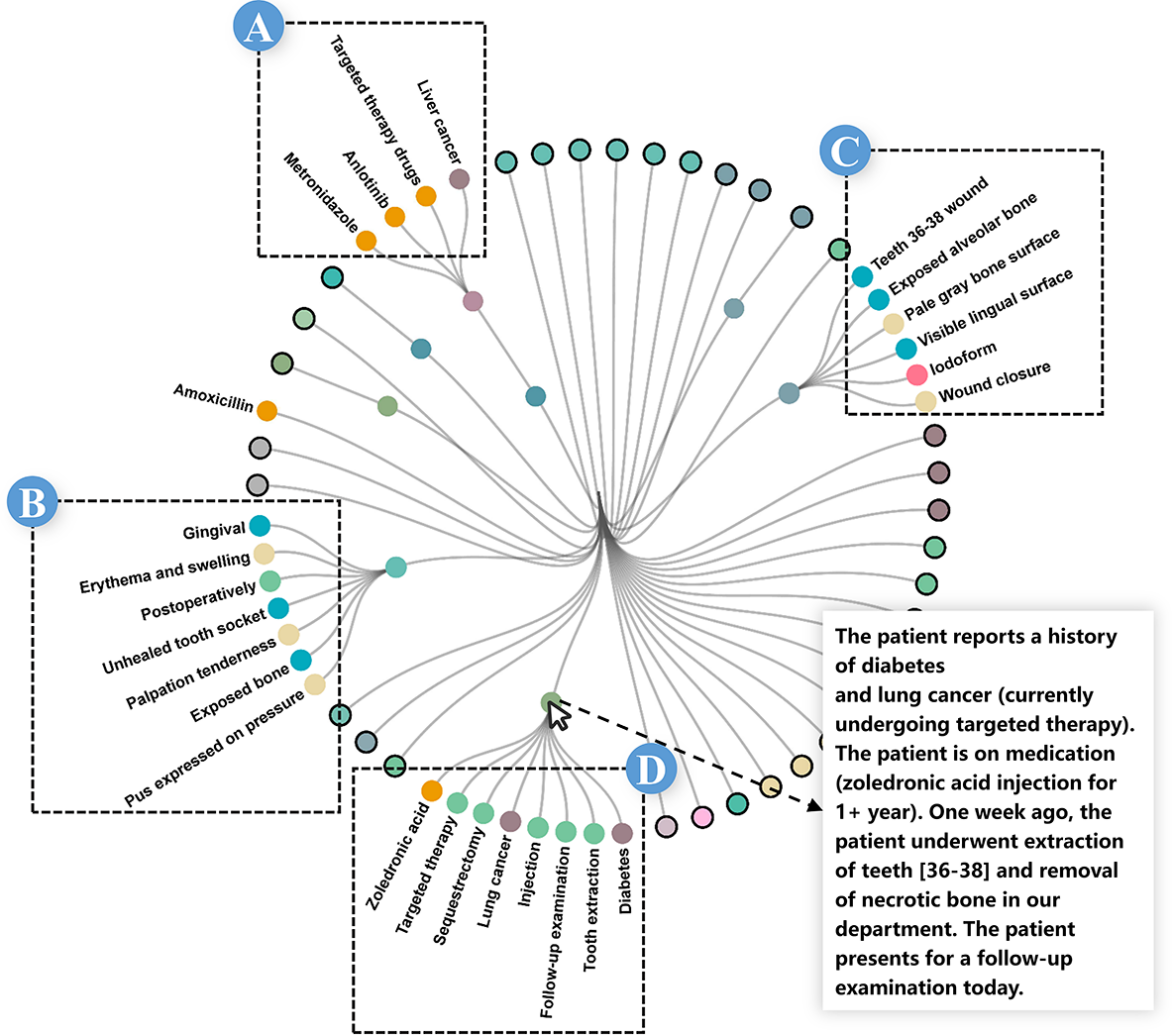
The association analysis view of the MES and MEs within the selected subgraph facilitates the exploratory analysis of relationships. ME: medical entity; MES: medical entity set.

The selected subgraphs were organized into the association analysis view (Figure 7). The experts first analyzed the MES (Figure 7A), which is distinguished by a high proportion of *dru* MEs based on its color encoding. Upon clicking to expand this MES, it was found to contain the *targeted therapy drugs*, *Anlotinib*, which is closely associated with the *liver cancer* ME, indicating the patient had a history of malignancy and related drug treatment. The text in Figure 7D further corroborated this by containing *lung cancer* and *targeted therapy*, noting specifically that the patient was using *zoledronic acid*. Therefore, E3 concluded that cancer status and related medications were factors associated with EC and FT. Regarding CQ, E3 analyzed the MES characterized by high *bod* and *sym* proportions (Figure 7B and C), identifying bone exposure and exudate as primary CQ factors. E3 concluded, “Performing association analyses on real-case factors can be integrated into clinical reasoning training, enhancing learners’ clinical analysis skills.”

#### Case 3: In-Depth Analysis and Construction of Illness Scripts

**Figure 8.**
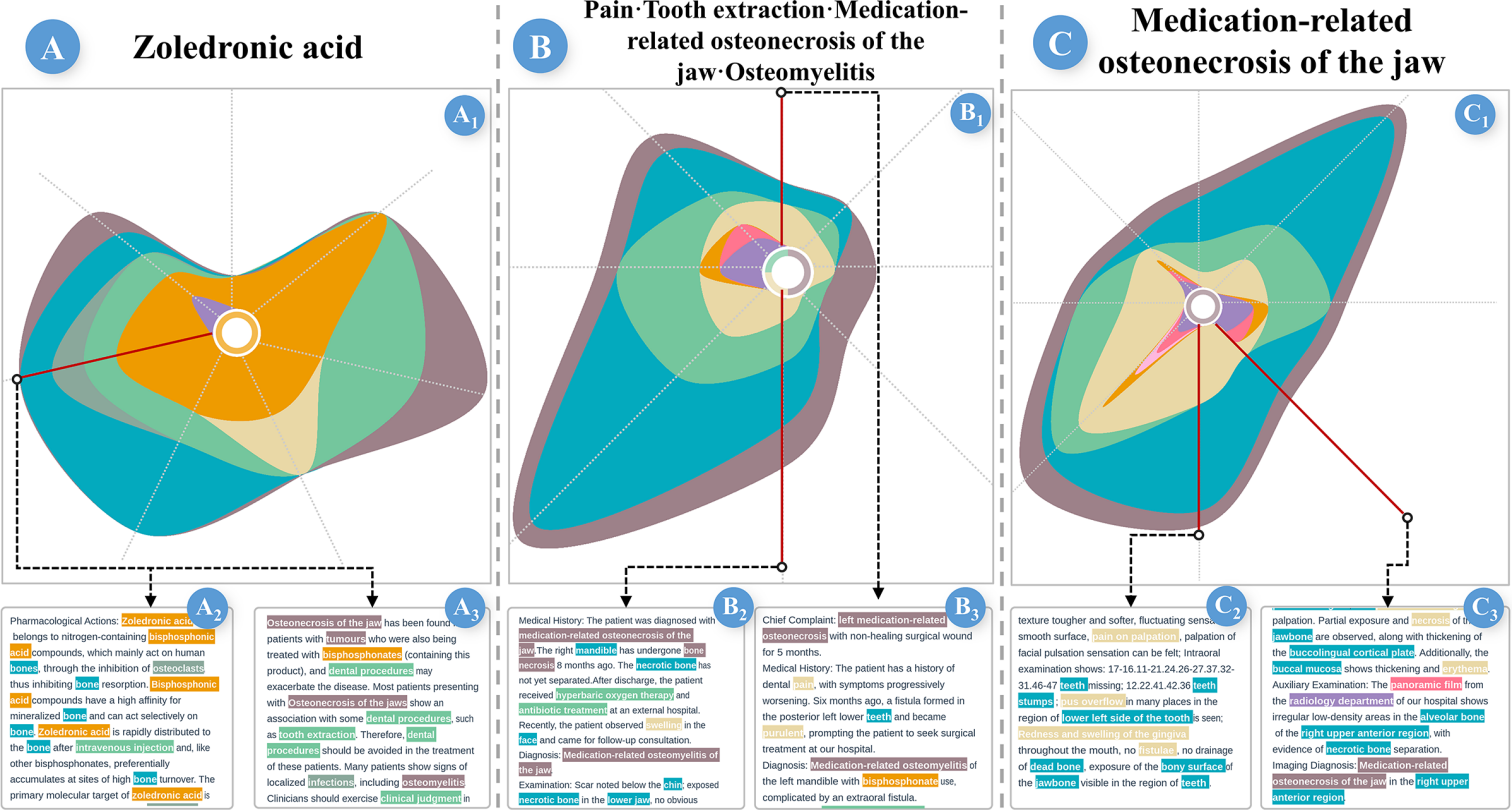
(A) Focused analysis of zoledronic acid. (B) Focused analysis of the medical history of MES, including tooth extraction. (C) Focused analysis of MRONJ. MES: medical entity set; MRONJ: medication-related osteonecrosis of the jaw.

To construct a comprehensive illness script, the experts began by analyzing the factors presented in case 2. E5 was initially interested in the role of *zoledronic acid* as an EC for the development of MRONJ, so he focused on it and generated a focused sectional view (Figure 8A1). The relevant MES includes a *mic* ME potentially related to the FT. A review of the corresponding MD (Figure 8A2) revealed that this ME pertains to *osteoclasts*, detailing the drug’s pharmacological action of inhibiting osteoclast growth, which may impair bone repair. The MD further indicates that when bisphosphonates are used in cancer patients, concurrent dental procedures such as tooth extraction may trigger MRONJ (Figure 8A3).

Subsequently, E3 examined a medical history MES involving *tooth extraction*, such MESs typically feature a relatively high density of *pro* and *dis* MEs (Figure 8B1). By examining the MDs of associated chief complaints and other medical history MESs (Figure 8B2 and B3), multiple co-occurring *pro* MEs were found to mention *osteomyelitis*, which was also referenced in Figure 8A3, suggesting that osteomyelitis may have developed as a CQ of MRONJ, likely secondary to an underlying infection.

To obtain a more definitive CQ, E5 performed a focus analysis of MRONJ (Figure 8C1), reviewing the MDs corresponding to the MESs that have *sym* and *equ* MEs (Figure 8C2 and C3). The analysis revealed that bone exposure and pus discharge were prominent clinical symptoms, whereas necrotic bone and radiolucent areas observed on panoramic radiographs emerged as distinctive imaging features. These findings can be used as reliable criteria for clinical diagnosis.

This analysis enabled the experts to construct a comprehensive illness script for MRONJ as follows:

- **EC:** Received treatment with bisphosphonates or related medications; a history of cancer or related systemic diseases; recent dental procedures.
- **FT:** Medications such as bisphosphonates inhibit osteoclast function, while dental procedures, particularly tooth extractions, can cause trauma, both of which contribute to the onset of MRONJ.
- **CQ:** Exposed bone, pus drainage, and potential for subsequent infections, such as osteomyelitis.

As E3 noted, “MExplore enables learners to construct illness scripts in a short period, facilitating rapid clinical reasoning and enhancing long-term retention of script knowledge.”

### User Study

**Figure 9.**
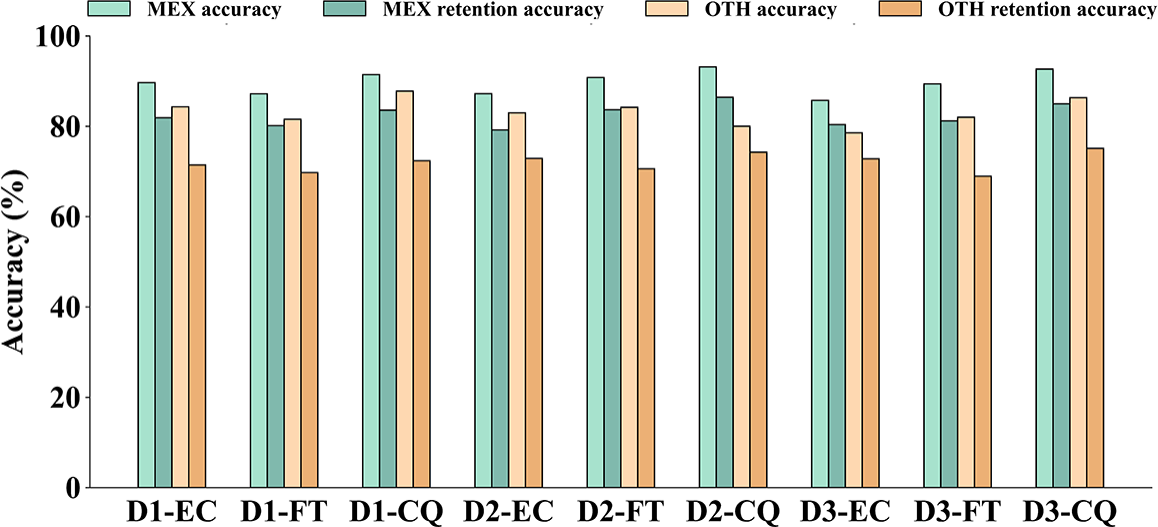
User study results: comparison of accuracy and retention accuracy. CQ: consequences; D1: oral candidiasis; D2: meningitis; D3: herpes zoster; EC: enabling conditions; FT: fault; MEX: experimental group; OTH: control group.

Tables5  6 present the mean accuracy and retention scores, whereas Table 7 provides the detailed completion time for each disease across various information classification methods. A more granular comparison of accuracy and retention performance is illustrated in Figure 9.

**Table 5.** User study accuracy score results.

	Max score	Accuracy score		
		MEX^a^, mean (SD)	OTH^b^, mean (SD)	*P* value
Oral candidiasis				
Enabling conditions	7	6.3 (0.48)	5.9 (0.57)	.11
Fault	7	6.1 (0.32)	5.7 (0.48)	.04
Consequences	8	7.3 (0.48)	7 (0.47)	.18
Meningitis				
Enabling conditions	7	6.1 (0.56)	5.8 (0.92)	.39
Fault	10	9.1 (0.32)	8.4 (0.52)	.002
Consequences	10	9.3 (0.67)	8 (0.47)	<.001
Herpes zoster				
Enabling conditions	9	7.9 (0.88)	7.1 (0.57)	.03
Fault	10	8.9 (0.57)	8.2 (0.42)	.006
Consequences	8	7.4 (0.52)	6.9 (0.57)	.05

a Acquiring expertise via MExplore.

b Acquiring expertise without MExplore but using other resources.

**Table 6.** User study retention accuracy score results.

	Max score	Retention accuracy score		
		MEX^a^, mean (SD)	OTH^b^, mean (SD)	*P* value
Oral candidiasis				
Enabling conditions	7	5.7 (0.67)	5 (1.05)	.09
Fault	7	5.6 (0.70)	4.9 (0.74)	.04
Consequences	8	6.7 (0.67)	5.7 (1.34)	.05
Meningitis				
Enabling conditions	7	5.5 (0.53)	5.1 (0.99)	.28
Fault	10	8.4 (0.70)	7.1 (0.88)	.002
Consequences	10	8.6 (0.84)	7.4 (0.97)	.008
Herpes zoster				
Enabling conditions	9	7.2 (1.03)	6.7 (1.06)	.30
Fault	10	8.1 (0.74)	6.9 (0.99)	.007
Consequences	8	6.8 (1.03)	6 (1.15)	.11

a Acquiring expertise via MExplore.

b Acquiring expertise without MExplore but using other resources.

**Table 7.** User study completion time results.

	Completion time (minutes)		*P* value
	MEX^a^, mean (SD)	OTH^b^, mean (SD)	
Oral candidiasis	10.45 (1.41)	12.32 (1.88)	.02
Meningitis	13.40 (1.73)	13.85 (2.41)	.64
Herpes zoster	11.82 (1.76)	15.05 (2.54)	.004

a Acquiring expertise via MExplore.

b Acquiring expertise without MExplore but using other resources.

The results showed that the MEX group demonstrated superior performance. Independent *t* tests confirmed significant improvements in overall mean accuracy (68.3 out of 76 (90%) vs 63.1 out of 76 (83%), *P*<.001). This advantage persisted in long-term retention: the MEX group achieved an overall mean retention accuracy of 62.5 out of 76 (82%), significantly surpassing the 54.7 out of 76 (72%) observed in the OTH group (*P*=.003). These findings suggest that the multilevel metaphors and interactive exploration in MEX may facilitate knowledge consolidation into long-term memory. Regarding efficiency, the MEX group had significantly shorter completion times for D1 (*P*=.02) and D3 (*P*=.004). Beyond speed, the MEX group exhibited consistently lower SDs across all tasks (Table 7), suggesting that the structured exploration path may reduce user disorientation and cognitive load when navigating complex medical knowledge.

### Expert Interview

**Figure 10.**
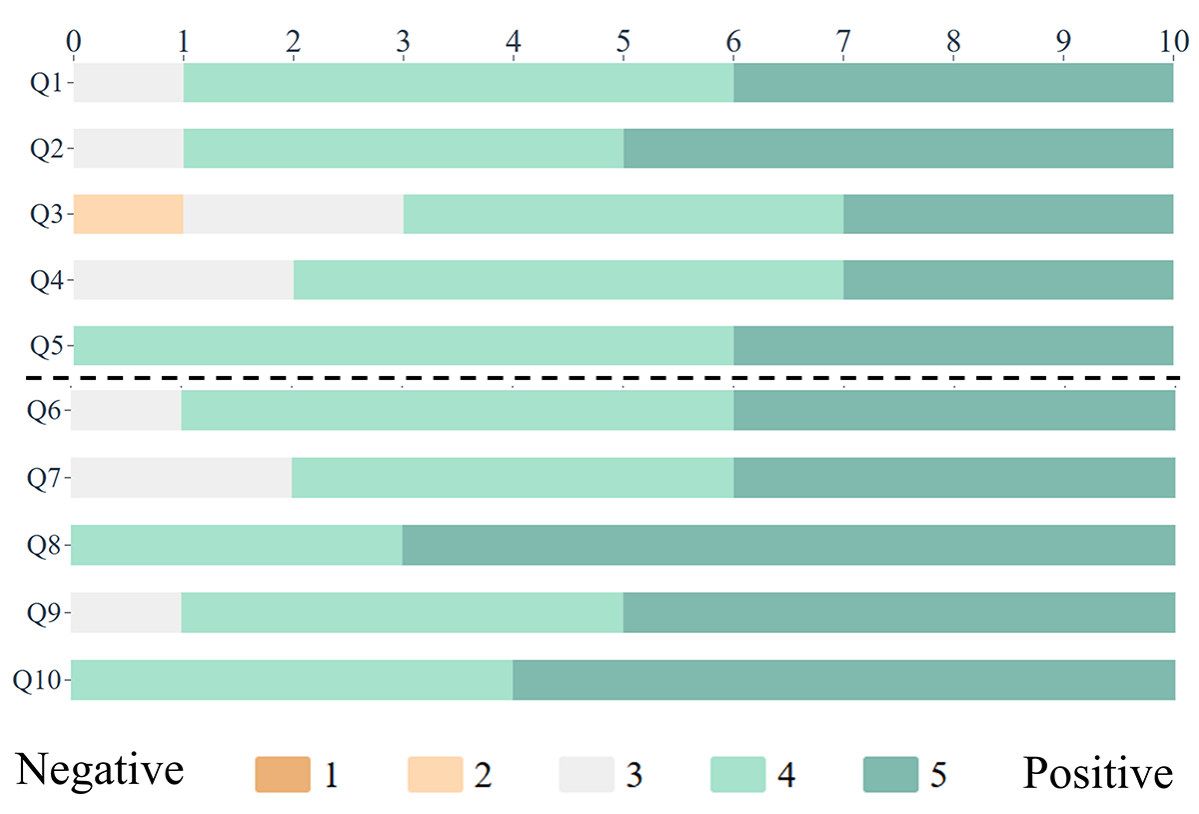
Results of the questionnaire. The number at the top represents the number of expert responses. The color of the bar represents the degree of negative (1 and 2), positive (4 and 5), or neutral (3) response.

The results of the questionnaire are presented in Figure 10.

#### System Performance

The experts all recommended MExplore for its user-friendly interface and interaction capabilities. The system’s hierarchical visualization, organized by data granularity and incremental exploration ability, significantly enhances user understanding and analysis. Although some of the experts (E5-E8) had no prior experience with visual analytics systems, they were able to easily comprehend and effectively use the system after receiving minimal training. E4 concurred that the ME-based exploration learning process in MExplore allows learners to focus on the core concepts of the target domain while creating an independent exploration path. E2 suggested incorporating features such as exploration playback and snapshot functionality to further increase the system’s usability. In summary, the experts highly praised the system’s visual design and interaction approach, asserting that it holds significant potential to improve the efficiency of knowledge acquisition for medical professionals.

#### Analysis Requirements

As shown in Figure 10, the experts believe that most of the key analytical requirements have been well met. E9 noted that the design of the association analysis view and the focused sectional view enabled users to correlate and concentrate their analysis on key knowledge points, thereby identifying relevant and valuable information—an essential aspect of the knowledge acquisition process. E8 noted that compared with search-based learning methods or chatbots, which have become popular in recent years, this approach can exercise the learner’s thinking ability and improve knowledge retention and accumulation. E5 further highlighted, “In addition to assisting novices in acquiring expertise, MExplore can also support experts and researchers in disease studies by aiding in research, summarization, and the discovery of new features.”

## Discussion

### Lessons Learned

The key insight from this study is the importance of comprehensibility. Learners must be able to focus on acquiring knowledge throughout the learning process, and the visualizations and interactions should be designed in a way that does not overwhelm or distract users. Through an iterative design process involving both experts and users, we find a clear preference for simple and intuitive visual forms. Based on this feedback, we developed a multilevel metaphor visual analytics framework to reduce cognitive strain and facilitate a shift in paradigm from traditional learning methods to more interactive forms of knowledge acquisition.

### Generalizability

#### Language Adaptability

Although MExplore uses a model pretrained on Chinese corpora, the framework is not strictly bound by linguistic characteristics. Transformer-based architectures have demonstrated robust concept understanding across diverse languages when fine-tuned on domain-specific data [76]. Consequently, the pipeline can support cross-lingual applications by incorporating domain-specific clinical corpora.

#### User Adaptability

As experts indicated in the Analysis Requirements section, MExplore’s extraction and knowledge association capabilities benefit a broad spectrum of users, not limited to novices. It can further assist senior clinicians and researchers in identifying novel disease features and advancing clinical studies.

#### Data Adaptability

To ensure broad generalizability, our retrieval and filtering mechanisms primarily leverage the semantic embedding similarity of unstructured text. However, we recognize that practical medical education often requires precise constraints. Our framework allows users to incorporate a deterministic metadata filtering layer (eg, filtering by visit date, department, and document type) into the search module tailored to their specific datasets. This enables the framework to meet curriculum-specific requirements and adapt to diverse educational goals beyond unstructured content analysis.

#### Domain Adaptability

The methodology can be extended beyond medicine to other domains requiring structured knowledge exploration, such as jurisprudence. By fine-tuning predictive models on annotated domain-specific texts, the MExplore framework can be repurposed to visualize entity relationships and facilitate knowledge acquisition in nonclinical fields.

### Limitations

A notable limitation is the linguistic specificity of the current dataset. Clinical documents in different languages exhibit unique syntactic complexities and sublanguages. While our pipeline is architecture-agnostic, performance in non-Chinese contexts depends on the availability of high-quality domain-specific corpora. Future iterations could incorporate neural machine translation and annotation projection to automatically generate training resources for low-resource languages, thereby enhancing multilingual applicability. Additionally, we plan to expand the dataset through multi-institutional collaboration to cover a broader range of diseases. While the system currently supports large-scale queries, further optimizations—such as approximate nearest neighbor search and WebGL rendering—will be explored to accommodate expanding data volumes. Lastly, the user study was conducted with a cohort of medical students (n=20). While suitable for testing learnability, this demographic may not reflect expert clinical decision-making. Future work will expand evaluations to practicing clinicians to better validate the system’s professional utility and generalizability.

### Conclusions

In this paper, we introduced MExplore, a visual analysis tool designed to support medical learners in exploring and acquiring medical knowledge. MExplore extracts MEs from large-scale medical texts and constructs a knowledge structure, offering a multilevel metaphor visual analytics framework. This framework includes 4 coordinated views: the MD space view, the MP star map, the association analysis view, and the focused sectional view. Together, these views enable users to tailor their learning paths, fostering deeper comprehension and improved retention. By facilitating the construction of complex, interconnected schemas typical of medicine, MExplore significantly supports learners in acquiring medical expertise.

We conducted 3 case studies, a user study with real-world datasets, and expert interviews. The results highlight MExplore’s effectiveness in facilitating the exploration and analysis of medical texts, significantly enhancing learning efficiency and supporting the acquisition of medical expertise.

## Abbreviations

BERT: Bidirectional Encoder Representations From Transformers
CBLUE: Chinese Biomedical Language Understanding Evaluation
CQ: consequences
EC: enabling conditions
FT: fault
LLM: large language model
LoRA: Low-Rank Adaptation
MD: medical document
ME: medical entity
MES: medical entity set
MEX: experimental group
MP: medical paragraph
MRONJ: medication-related osteonecrosis of the jaw
MWCSS: Medical Text of West China School (Hospital) of Stomatology
NER: named entity recognition
OTH: control group
QLoRA: Quantized Low-Rank Adaptation
